# The growth, development and infection process of the plant pathogen *Fusarium*

**DOI:** 10.1080/15592324.2025.2573097

**Published:** 2025-11-02

**Authors:** Zhuo Li, Fengying He, Xiaotong Gai, Hongyao Zhu, Shidao He, Yuan Hu Xuan, Luchao Bai

**Affiliations:** a Department of Plant Protection, State Key Laboratory of Elemento-Organic Chemistry, National Pesticide Engineering Research Center (Tianjin), Nankai University, Tianjin, China; b College of Agriculture and Animal Husbandry, Qinghai University, Xining, China; c Yunnan Academy of Tobacco Agricultural Sciences, Kunming, China; d Institute of Tropical Bioscience and Biotechnology, Chinese Academy of Tropical Agricultural Sciences, Haikou, China

**Keywords:** *Fusarium*, growth, development, A-to-I editing, pathogenicity

## Abstract

The genus *Fusarium* includes some of the most detrimental pathogenic fungi to crops, significantly impacting cereal growth and food production. It causes devastating diseases such as banana wilt caused by *Fusarium oxysporum* and Fusarium head blight caused by *Fusarium graminearum*. Beyond causing substantial yield losses, *Fusarium* species produce various mycotoxins that pose serious risks to crop safety and human health. Recent advances in genome sequencing have uncovered numerous genes involved in secondary metabolism, hyphal development, reproduction, and virulence mechanisms in *Fusarium*. This review summarizes current knowledge on the growth, development, and pathogenesis of the *Fusarium*, with a focus on *F. oxysporum* and *F. graminearum*. Elucidating these mechanisms is crucial for developing targeted fungicides and innovative management strategies to control Fusarium diseases, thereby reducing their agricultural and health impacts.

## Background

*Fusarium*, a filamentous ascomycete fungus belonging to the *Nectriaceae* family within the Sordariomycetes order Hypocreales, encompasses many toxin-producing plant pathogens of agricultural importance.^1^ These pathogens contribute to a range of plant diseases in agricultural and natural environments, manifesting as wilts, blights, rots, and cankers.^2^ The economic impact of plant diseases caused by *Fusarium* on agriculture worldwide amounts to billions of USD annually.^3^ Mycotoxins, which are toxic secondary metabolites produced by *Fusarium*, serve as virulence factors in plant diseases and can contaminate agricultural products, rendering them unfit for consumption.^4^ Moreover, *Fusarium* spp. pose significant health risks to humans, especially immunocompromised individuals, by causing a spectrum of infections that range from onychomycosis and keratitis, to pneumonia, fungemia, disseminated disease, and rare invasive manifestations such as sinusitis, osteomyelitis, and endophthalmitis.^5^ On the other hand, *Fusarium* has emerged as one of the most prevalent endophytic fungal genera, producing diverse bioactive secondary metabolites.^6^

This review focuses on growth and development, and pathogenesis of the plant pathogen *Fusarium*. This comprehensive analysis sheds new light on disease control strategies and plant resistance breeding schemes by providing valuable insights into the growth, development and pathogenic mechanisms of *Fusarium*.

## Plant diseases caused by *Fusarium*

In the past two decades, the application of phylogenetic species recognition based on genealogical concordance and nondiscordance (GCPSR) has led to the identification of approximately 400 phylogenetic species of *Fusarium.*^1^ However, the majority of these phylogenetic species remain unnamed due to their cryptic morphology.^7^ Historically, research on *Fusarium* taxonomy has focused primarily on the asexual morph (anamorph), which is more commonly encountered in nature by biologists, whereas the sexual morph (teleomorph) is largely unknown for most species.^2^

A recent study revealed that, on the basis of phylogenetic analysis of exonic nucleotide sequences of 19 orthologous protein-coding genes, 23 clades within the monophyletic *Fusarium* genus were identified as species complexes, encompassing all economically significant groups.^8^ Among these, four main species complexes are known for their roles as plant pathogens: the *F. fujikuroi* species complex (FFSC), associated with bakanae disease of rice and ear rot of maize, and several species produce fumonisins (FUMs); the *Fusarium graminearum* species complex (FGSC), including wheat and barley pathogens that synthesize trichothecenes (Tris); the *Fusarium oxysporum* species complex (FOSC), which includes the species that can cause vascular wilt; and the *F. solani* species complex (FSSC), comprising various foot and root rot pathogens.^9^ A review of the list of agricultural and horticultural plant diseases on the American Society for Plant Pathology website (APS Home) revealed that more than 81 of the 101 economically vital plants mentioned were affected by at least one disease caused by *Fusarium.*^7^ These findings underscore the substantial influence of *Fusarium* on agricultural production. The diseases caused by *Fusarium* fungi can vary in severity and may manifest as root or stem rot, wilt, fruit or seed rot, or leaf diseases. The different types of crop diseases attributed to *Fusarium*, as documented in the literature, are summarized in Table 1.

**Table 1.** Plant diseases and symptoms caused by different fungi of the *Fusarium* spp.

Disease	Host	Pathogen	Reference	Symptomatic
Fruit rot	Banana	*F. acuminatum; F. moniliform; F. oxysporum; F. proliferatum; F. solani; F. subglutinans*	[10]	Rotting of the flesh, formation of brown lesions, and sometimes mycelia appear in the rot lesions.
	Papaya	*F. acuminatum*	[11]	
		*F. oxysporum; F. nivale*	[12]	
		*F. semitectum; F. solani*	[13]	
	Pineapple	*F. ananatum; F. concentricum; F. fujikuroi; F. guttiforme; F. incarnatum; F. oxysporum; F. polyphialidicum;* *F. proliferatum; F. temperatum; F. verticillioides; F. proliferatum; F. ananatum*	[14]	
	Avocado	*F. semitectum; F. equiseti; F. graminearum*	[15]	
	Winter squash	*F. sambucinum*	[16]	
	Pumpkin	*F. tricinctum*	[17]	
	Cherry	*F. equiseti; F. fujikuroi; F. lateritium; F. proliferatum; F. acuminatum*	[18]	
Fusarium wilt	Peanut	*F. oxysporum*	[19]	Mature leaves turn yellow and wilted, the disease then progresses to the younger leaves that surround the pseudostem.
		*F. solani*	[20]	
	Potato	*F. oxysporum; F. equiseti; F. redolens; F. acuminatum*	[21]	
	Tomato	*F. oxysporum; F. andiyazi; F. circinatum*	[22]	
	Strawberry	*F. oxysporum* f. sp. *fragariae (Fof)*	[23]	
	*Pennisetum sinese*	*F. oxysporum*	[24]	
	Cotton	*F. oxysporum* f. sp. *vasinfectum (Atk.)*	[25]	
	Tea	*F. fujikuroi; F. solani; F. oxysporum; F. concentricum*	[26]	
	Marijuana	*F. solani; F. proliferatum*	[27]	
	Banana	*F. oxysporum f.sp. cubense*	[28]	
	Avocado	*F. oxysporum; F. solani; F. equiseti*	[29,30]	
Root rot	Peanut	*F. solani*	[31]	Root and stem rot, as well as rotting and wilting of young plants.
	Papaya	*F. solani*	[32]	
		*F. falciforme*	[33]	
	Kidney bean	*F. cuneirostrum*	[34]	
	Soybean	*F. proliferatum*	[35]	
	*Panax notoginseng*	*F. oxysporum; F.solani*	[36]	
	Sugar beet	*F. oxysporum*	[37]	
	Tobacco	*F. sacchar; F. oxysporum; F. solani; F. verticillioides*]	[38]	
	Soybean	*F. graminearum; F. acuminatum; F. oxysporum; F. armeniacum; F. proliferatum; F. virguliforme;* *F. solani species complex; F. commune; F. scirpi; F. clavum; F. acuminatum; F. avenaceum;* *F. sporotrichioide*	[39]	
Leaf spot	Peanut	*F. iomoeae*	[40]	Discolored lesions or spots on the leaves caused by necrosis of the tissues.
	Tomato	*F. proliferatum*	[41,42]	
	Mango	*F. Proliferatum; F. semitectum; F.chlamydosporum*	[42]	
		*F. Verticillioides; F. mangiferae; F. pernambucanum; F. Proliferatum; F. sulawesiense; F. concentricum* *F. hainanense*	[43]	
	Pineapple	*F. oxysporum; F. solani; F. proliferatum; F. verticillioides,*	[44]	
	*Cymbidium* orchids	*F. subglutinans*	[45]	
	Tobacco	*F. proliferatum*	[46]	
	Cherry	*F. Luffae; F. lateritium; F.compactum; F. nygamai; F. citri; F. ipomoeae; F. curvatum*	[47]	
	Spinach	*F. equiseti*	[48]	
	Kiwifruit	*F. graminearum.*	[49]	
	*Rehmannia glutinosa*	*F. equiseti; F. acuminatum*	[50]	

## The reproduction process of *Fusarium*

In the *Fusarium* genus, the reproductive modes of different species are diverse. For example, *F. oxysporum* relies mainly on asexual reproduction (propagating through conidia), *F. graminearum* completes the sexual cycle through homothallic reproduction (self-compatible sexual reproduction),^51^ and *F. fujikuroi* uses heterothallic reproduction (sexual reproduction that requires the combination of different mating types).^52^ This difference in reproductive strategies reflects the diverse choices of the *Fusarium* genus in the evolutionary process for environmental and host adaptability. At the beginning of the sexual reproduction of *F. graminearum*, part of the asexual mycelium differentiates to form the antheridium and the ascogonium, and after contact, plasmogamy results in the formation of two nucleated cells.^53^ At the same time, the antheridium will form an ascophore after contact with the ascogonium. Next, the binuclear cells at the tip of the ascophore elongate and bend to form crozier cells.^54^ Approximately 24 h later, the initial ascus and ascus mother cells begin to form. The binuclear cells in croziers continue to divide into four nuclei and form two monocytes as well as one binuclear cell, the ascus mother cell.^55^ Ascus mother cells eventually produce 8 ascus spores and develop into ascus through karyogamy, meiosis, and mitosis.^56,57^ Ascospore formation is the last step of sexual reproduction. When the ascospore matures, the pressure inside the ascospore causes the air ejected from the apex orifice of the ascospore to infest the wheat again.^53^ *Fusarium* can produce three types of asexual spores: macroconidia, microconidia, and chlamydospores.^53^ Macroconidia are scattered on aerial hyphae or conidiophores, exhibiting diverse shapes including clavate, falcate, elongated tubular, and fusiform forms, with 3 or 4 septa. Differences in the shape of the macroconidia are central to the identification of many *Fusarium* species.^53^ Microconidia are predominantly unicellular, with a few exhibiting 1−3 septa. The most common shapes of microconidia are: oval, kidney shaped, obovoid, pyriform, napiform, globose, and fusiform. Chlamydospores are spherical with a thick wall. And chlamydospores are predominantly produced in a merotrophic manner from hyphae, or directly transformed from hyphae or spore cells after altering their cellular contents density. Chlamydospores may be formed singly, doubly, in clumps and in chains.^53^ They often take a long time to produce, and may not be produced in large numbers. These spores can survive for years in a dormant state within soil or humus, and chlamydospores in soil are extremely difficult to eradicate.^53^

**Figure 1.**
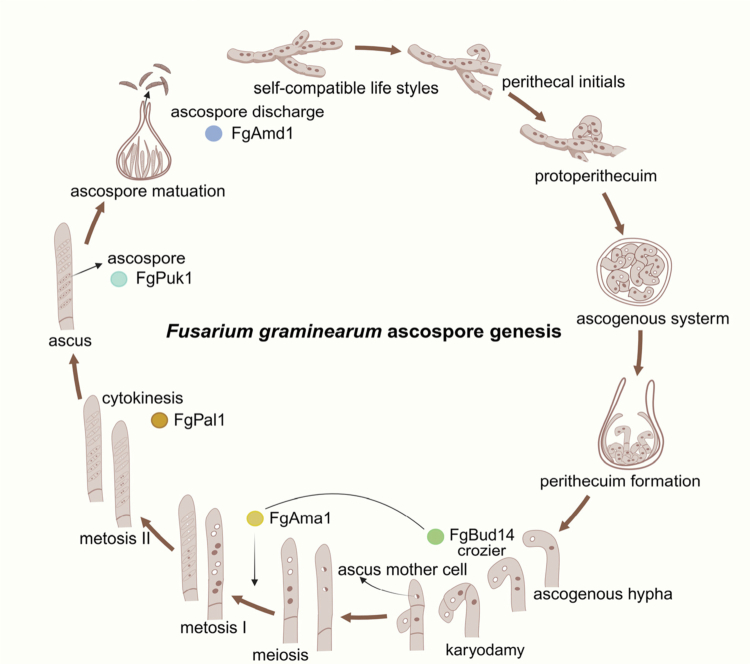
Life cycle of *F. graminearum*. The colored circles indicate the stages at which different genes involved in A-to-I RNA editing play a role in the sexual reproduction of *F. graminearum*. A-to-I RNA editing in *F. graminearum* occurs in the nucleus to affect the proteins (FgPal1, FgAma1, FgAmd1, and FgPuc1) responsible for PSC events and regulate the formation of ascospores in *F. graminearum*.

Adenosine-to-inosine (A-to-I) RNA editing catalyzed by adenosine deaminase acting on RNA (ADAR) enzymes occurs specifically during sexual reproduction in *Fusarium* genus.^58^ RNA editing may play an important role in regulating the functions of genes that are crucial for ascospore formation or the forcible discharge of ascospores (Figure 1). It can re-encode *FgPUK1* (*perithecium unique kinase 1*), *FgAMA1* (*apical membrane antigen 1*), *FgPAL1* (*pears and lemons*), *FgAMD1* (*ascus maturation and ascospore discharge 1*) and other genes, resulting in premature codon stop (PSC) events.^58‐61^ *PUK1* is a type of gene specifically expressed during sexual reproduction. The orthologous genes of *PUK1* in *F. graminearum* and *F. verticillioides* undergo A-to-I editing at the same sites.^60^ The open reading frame of PUK1 has two stop codons UAG, which are tandemly prematurely terminated through A-to-I RNA editing, playing an important role in the development and release of ascospores. In addition, most of the orthologous genes involved in *PUK1*-like editing events in *F. graminearum* also have similar editing sites in *F. verticillioides.*^60^ The conidial morphology of the *Fgpuk1* mutant was normal. However, during late sexual reproduction of *F. graminearum,* the *Fgpuk1* mutant could form a normal ascus but could not produce four-cell ascus spores normally. The number of ascospores is reduced to single or double cells that are spherical or fragmented.^60^ Similarly, as with *FgPUK1*, A-to-I RNA editing in the PSC functional region of *FgAMA1* is important for sexual reproduction in *F. graminearum*. With the deletion of the *FgAMA1* gene, meiotic division was normal, and 8 ascospores could still be produced, but the ascospore morphology was abnormal in the state of a single cell and two nucleons. These findings suggest that *FgAMA1* plays an important role in cytoplasmic division and the second round of mitosis during ascospore development.^59^ A protein located at the tip of croziers, FgBud14, interacts with FgAma1 and plays an important role in crozier formation, ascus development, and ascus spore formation.^62^ In addition, *FgPAL1* is affected by *FgAMA1* deletion and upregulated in *Fgama1* mutants, and its PSC also has an A-to-I editing event. In contrast to *Fgpuk1* mutants and *Fgama1* mutants, *Fgpal1* mutants normally produce eight ascospores but exhibit severe growth defects during ascospore formation, and ascospore development is often aborted, resulting in failure to form ascospores.^61^ Another gene, *FgAMD1*, which is edited from A-to-I to encode a functional full-length protein, is located in the ascus membrane and plays a key role in maintaining the integrity of the ascus wall during maturation.^58^ Owing to the premature degradation of the ascus wall of the *Fgamd1* mutant, the ascus shell could not discharge the ascus spores normally, thus obstructing the sexual reproduction process of *F. graminearum.*^58^ Surprisingly, the lack of *FgAMD1* results in increased expression of numerous genes associated with transporter activity and membrane function. The average level of A-to-I RNA editing in *F. graminearum* is similar to that in animals,^63^ indicating that such editing may play an important role in the regulation of protein function adaptability and diversity during the sexual reproduction stage of *F. graminearum.*^64^ This mechanism may help fungi optimize reproductive strategies and enhance genetic plasticity in complex environments or host interactions by dynamically regulating the coding sequences of key genes.

## Infection and colonization of host plants by *Fusarium*

Common modes of *Fusarium* transmission include vertical and horizontal spread. Vertical transmission occurs through maternal inheritance to the next generation of seeds, while horizontal transmission refers to pathogens in soil and diseased plant debris infecting hosts through wounds, causing plant disease.^65^ And *Fusarium* utilizes various infection strategies, most of which fall into the category of hemibiotrophs. Initially, infection resembles pathogen dependence on a living host (biotrophic) but eventually transitions to killing and consuming host cells (necrotic).^2^ *Fusarium* diseases can manifest in roots through soil-borne inoculum or in above-ground plant parts through air or water dispersal.^65^ The spores of *F. oxysporum* germinate into germ tubes that can directly penetrate the epidermal cells of lateral roots or enter through tiny wounds. Upon penetration, an appressorium structure is formed at the entry point.^66‐68^ The fungus then rapidly grows within the host cell, branching out into a network of hyphae.^67^ Interestingly, *F. oxysporum* can penetrate and grow inside host cells without causing severe damage, such as plasma lysis or cell necrosis, suggesting a biotrophic phase during root tip colonization^68^. Eventually, the fungus advances into the root cortex, colonizing vascular tissue and leading to wilting, necrosis, and chlorosis in above-ground plant parts.^69^ As a social phenomenon, *Fusarium* plant diseases have several major impacts. One of these was the near-devastating destruction of the commercial banana industry in the 1960s by Panama wilt caused by *F. oxysporum* f. sp. *cubense* (Foc). Today, global banana production continues to face a threat from Tropical Race 4 (TR4).^70^ Moreover, rice bakanae disease caused by *F. fujikuroi* can reduce crop yields ranging from 30% to 95%, resulting in considerable economic losses.^71^

In contrast, *F. graminearum*, the major cause of Fusarium head blight (FHB) in cereals worldwide, causes limited necrosis.^2^ During wet or windy conditions in the wheat flowering phase, conidia or ascospores of *F. graminearum* attach to the wheat ear surface or enter open glumes. Following germination, hyphae form and proceed to infect cells, growing both intracellularly and intercellularly.^72,73^ The mycelium subsequently advances into the ovary, rachis, and neighboring spikelets via the vascular bundle, leading to disease symptoms that ultimately destroy the grain and contaminate it with toxins.^74^ The impact of climatic conditions on *Fusarium* infection in temperate regions is primarily reflected in environmental factors such as temperature, humidity, and rainfall. Studies have indicated that temperature and humidity are key factors influencing Fusarium growth and mycotoxin production.^75‐77^ In temperate countries, precipitation during anthesis drives *Fusarium* epidemics through a triple mechanism: rain splash and air turbulence disperse spores to floral organs^78,79^; high humidity maintains a water film on the anther surface, supporting spore germination and the differentiation of infection structures within 6−12 h^73^; precipitation events at 15 °C−30  °C coinciding with wheat anthesis provide a competitive advantage to highly virulent species such as *F. graminearum.*^76^ Altered precipitation patterns drive pathogen population evolution. Climate change has increased rainfall in temperate regions, promoting a shift toward more moisture-tolerant and aggressive species. Over the past two decades, Northern Europe has seen a transition from the cool-adapted *F. culmorum* to the warm-adapted *F. graminearum*, which produces higher toxin levels and exhibits greater aggressiveness.^76^ Under moist conditions, *F. graminearum* synthesizes more deoxynivalenol (DON), which further suppresses host defense responses and accelerates mycelial spread within the rachis.^80^,^73^ Factors such as global warming, reduced tillage, crop rotation technologies and straw recycling, severe FHB epidemics have occurred at least every 4 or 5 y.^94^ The average yearly occurrence of FHB in China affects more than 4.5 million hectares, approximately 20% of the total planted area of wheat, and has caused annual yield losses of more than 3.41 million tons from 2000−2018.^89^ In the United States, wheat and barley losses from scab epidemics in the 1990s approached $3 billion, compelling changes in cropping practices and forcing many family farms out of business.^85^

## Conclusion

*Fusarium* spp. is projected to retain substantial biological and socioeconomic relevance in the coming decades, exacerbated by climate-driven increases in phytopathogenicity and mycotoxin risks. While chemical fungicides remain widely deployed, their prolonged use has accelerated fungal resistance evolution, necessitating innovative control strategies. Critical challenges persist in managing *F. oxysporum*-induced vascular diseases, where soil microbiome modulation through microbial community engineering showed promising outcome in establishing pathogen-suppressive rhizosphere environments.^92^ Combating pathogens like *F. graminearum* requires integrated approaches combining mycotoxin profiling, biosynthetic gene cluster analysis, and phylogenomic insights.^85,90^

Advances in *Fusarium* whole-genome sequencing and gene knockout techniques have revealed numerous functional genes associated with secondary metabolite production, hyphal differentiation, reproduction, and pathogenicity.^88,89^ Nevertheless, a significant proportion of genes remain unnoticed in existing databases, highlighting the need for further research. While various transcriptional regulatory factors and signaling pathways have been linked to the pathogenicity of *Fusarium*, the underlying mechanisms require further investigation.^93^ In particular, recent findings underscore the crucial role of epigenetic regulation in the growth, reproduction and pathogenesis of fungi.^83,84,92^ Understanding these mechanisms depends on the comprehensive integration of multiomics approaches such as transcriptomics, proteomics and metabolomics. Innovative technologies such as host-induced gene silencing (HIGS) or spray-induced gene silencing (SIGS) offer promising strategies for preventing and controlling diseases caused by *Fusarium.*^82,87,91^ For example, interfering with the signal transduction of *Fusarium* blocks the transition between saprophytic and pathogenic growth.

For the utilization of plant resistance genes, we can identify plant disease resistance genes through other pathological systems or capture and identify resistance and susceptibility genes in the host through the secretion of *Fusarium* protein as bait, thereby supporting the breeding of plant disease resistance.

Finally, we summarize the emerging trends, challenges, and future research directions of the plant pathogenic fungus *Fusarium* in the study.

1. In-depth dissection of pathogenic molecular mechanisms and signaling pathways: foundations for novel fungicide development.
2. Innovative applications of crop disease resistance breeding via e.g. gene editing technologies.
3. Integration of microbiome, metabolomics, and synthetic biological technologies for designing high-efficiency microbial-chemical synergistic control systems.
4. Investigating *Fusarium* adaptation mechanisms to climate change.
5. Development of AI- and big data-driven early warning systems for *Fusarium*-related diseases.
6. Optimizing international collaboration and integrated management strategies for *Fusarium* control.

*Fusarium* research is advancing into a new stage characterized by the integration of interdisciplinary approaches and technological innovations. Through analyzing molecular mechanisms, developing intelligent tools, optimizing biological control strategies, and strengthening global collaborations, the field is poised to transition from passive disease response to active intervention in prevention and control. These advances will not only ensure food security and safety but also promote the sustainable development of agricultural ecosystems.
